# Identification and Characterization of Novel Inhibitors of Human Poly(ADP-Ribose) Polymerase-1

**DOI:** 10.3390/molecules30132728

**Published:** 2025-06-25

**Authors:** Ibrahim Morgan, Robert Rennert, Robert Berger, Ahmed Hassanin, Mehdi D. Davari, Daniela Eisenschmidt-Bönn, Ludger A. Wessjohann

**Affiliations:** Department of Bioorganic Chemistry, Leibniz Institute of Plant Biochemistry, Weinberg 3, 06120 Halle (Saale), Germany; ibrahim.morgan@ipb-halle.de (I.M.); ahassanin@ipb-halle.de (A.H.); mehdi.davari@ipb-halle.de (M.D.D.); daniela.eisenschmidt-boenn@uk-halle.de (D.E.-B.)

**Keywords:** TNBC, PARP, cancer, cytoxicity, olaparib, synergism, SLFN

## Abstract

Poly(ADP-ribose) polymerases (PARP) are a family of enzymes that were proven to play an essential role in the initiation and activation of DNA repair processes in the case of DNA single-strand breaks. The inhibition of PARP enzymes might be a promising option for the treatment of several challenging types of cancers, including triple-negative breast cancer (TNBC) and non-small cell lung carcinoma (NSCLC). This study utilizes several techniques to screen the compound collection of the Leibniz Institute of Plant Biochemistry (IPB) to identify novel hPARP-1 inhibitors. First, an in silico pharmacophore-based docking study was conducted to virtually screen compounds with potential inhibitory effects. To evaluate these compounds in vitro, a cell-free enzyme assay was developed, optimized, and employed to identify hPARP-1 inhibitors, resulting in the discovery of two novel scaffolds represented by compounds **54** and **57**, with the latter being the most active one from the compound library. Furthermore, fluorescence microscopy and synergism assays were performed to investigate the cellular and nuclear pathways of hPARP-1 inhibitor **57** and its potential synergistic effect with the DNA-damaging agent temozolomide. The findings suggest that the compound requires further lead optimization to enhance its ability to target the nuclear PARP enzyme effectively. Nonetheless, this new scaffold demonstrated a five-fold higher PARP inhibitory activity at the enzyme level compared to the core structure of olaparib (OLP), phthalazin-1(2*H*)-one.

## 1. Introduction

Based on the Global Cancer Statistics 2022, close to 20 million cancer patients were newly diagnosed, of which approximately 2.3 million were females with breast cancer. Breast cancer is considered the most frequently occurring cancer type in females, with an incidence of 23.8% of all newly diagnosed patients, followed by lung and colorectal cancer, with incidence rates of 9.4% and 8.9%, respectively [1]. Breast cancers can be classified into different types based on the receptors expressed by the cancer cells, which are either hormone receptor-positive cancer cells (expressing estrogen and/or progesterone receptors), HER2/neu-positives (expressing erbB-2 receptors), or triple-negative breast cancer cells (TNBC, lacking the aforementioned receptors) [2]. The disease’s prognosis as well as the selection of the optimal therapy are highly dependent on the type of breast cancer. Estrogen receptor-positive breast cancers can be treated, for instance, with either estrogen receptor modulators, such as tamoxifen, or aromatase inhibitors, such as anastrozole [3,4]. The HER2/neu-positive breast cancer type can be treated with monoclonal antibodies like trastuzumab or more advanced HER2-targeting antibody–drug conjugates [5,6]. However, the most challenging breast cancer type is TNBC, which accounts for 15–20% of newly diagnosed breast tumors [7]. Due to the lack of the aforementioned standard breast cancer-associated receptors, the targeted treatment of TNBCs demands alternative molecular targets. Amongst those, the poly(ADP-ribose) polymerase enzyme could play a vital role in the treatment of TNBC tumors, but also as a prognostic marker [8].

Poly(ADP-ribose) polymerases (hPARP) are a family of proteins comprising 17 members in humans sharing a conserved catalytic domain [9]. This family is characterized by the ability to add either a single (mono) or several (poly) ADP-ribose molecule(s) to target proteins, a posttranslational modification (PTM) that plays a significant role in the regulation of the modified proteins’ function and activity [10]. In that sense, hPARP-1 is considered to be responsible for 80–90% of PARylations taking place in human cells [11]. The enzyme consists of six domains, namely the Zn1, Zn2, Zn3, BRCT, WGR, and CAT domains. The catalytic domain (CAT) consists of two subdomains known as helical subdomain (HD) and an ADP-ribose transferase (ART) subdomain [12]. Upon the occurrence of DNA strand breaks, the zinc finger domains (Zn1, Zn2) recognize and bind the DNA, leading to further interaction of Zn3 and WGR domain with the damaged DNA [13]. These interactions activate the hPARP-1 enzyme caused by a conformational change in the HD, increasing the ART domain’s exposure to NAD^+^ molecules [14]. NAD^+^ binds to the ART domain through a His-Tyr-Glu (H-Y-E) triad by H-bonding and ring stacking, which then acts as a donor for an ADP-ribose unit to initiate PARylation at an acidic amino acid moiety of the acceptor protein, followed by the elongation or branching of the PAR chain through the formation of 2′,1″-*O*-glycosidic ribose-ribose or 2″,1‴-glycosidic bonds, respectively [15]. In the context of DNA repair, the PARylation of hPARP-1 triggers the protein-protein interaction between the BRCA1 C-terminal (BRCT) domains of hPARP-1 and the cross-complementing protein 1 (XRCC1) [16]. Subsequently, the XRCC1 protein acts as a scaffold for DNA ligase III-α and DNA polymerase-β, which repair the DNA through either base or nucleotide excision repair mechanisms, depending on the type of the DNA damage [17].

hPARP was classified as an essential protein that could be chemotherapeutically targeted for the treatment of several cancer types, including more difficult-to-treat cancers such as TNBC or non-small cell lung carcinoma (NSCLC) [18,19]. As previously mentioned, hPARP’s main function is to assist the DNA damage repair. Hence, upon the exposure of tumor cells to DNA-damaging factors such as therapeutic radiation or chemotherapeutics like temozolomide (TMZ), the inhibition of hPARP can cause the failure of DNA repair and therefore tumor cell death [20,21]. However, that promising effect was mainly noticed in cancer types that have a mutated breast cancer gene (BRCA). That protein family is responsible for DNA repair through homologous recombination, [22] a tumor suppressor mechanism in healthy cells. However, in BRCA-positive tumor cells it acts as an additional escape and resistance mechanism against DNA-targeting chemotherapy [23]. Conversely, a synergistic interaction of hPARP inhibition and BRCA deficiency of tumor cells can permit a selective targeting of cancer cells while avoiding severe side effects on healthy cells, according to a concept known as synthetic lethality [24]. Additionally, a new family of proteins named Schlafen (SLFN) was acknowledged for being crucial for hPARP activity, especially SLFN-11, and was found to be a predictive biomarker for hPARP inhibitor-sensitive cancer types [25,26]. SLFN-11 plays an essential functional role in DNA repair as well. It is a helicase protein that is recruited to the stressed replication fork leading to replication blockage and finally cell death. In other words, cancer cells that are characterized by high SLFN-11 expression are predicted to be more sensitive to hPARP inhibitors [27].

**Figure 1:**
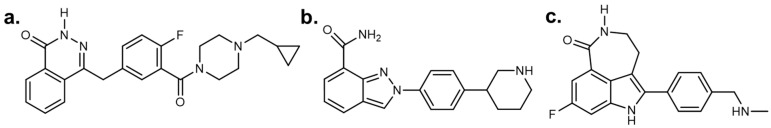
Structures of (**a**) olaparib (OLP), (**b**) niraparib, and (**c**) rucaparib.

hPARP inhibitors (hPARPi) are compounds that occupy the ART subdomain of the hPARP enzyme through interaction with Tyr907, Ser904, and Gly863, preventing the interaction and binding of NAD^+^ with the H-Y-E triad, which leads to failure of the PARylation process and, finally, DNA repair [28,29]. Moreover, the inhibition traps the hPARP enzyme to the damaged DNA, preventing it from detecting any further damaged DNA locus [30]. Due to these promising effects, several hPARPi were developed—including olaparib (OLP), rucaparib, and niraparib (Figure 1)—and are already approved for clinical use, either as a standalone medication or as an adjuvant for DNA damaging chemotherapeutics like TMZ [21,31]. In homologous recombination-deficient cancer, a correlation of 88.9% was found between the effectiveness of olaparib as a monotherapy and the deficiency of homologous recombination [32]. This means that hPARPi can clearly help to improve the treatment and prognosis of cancer patients, and therefore are indeed a very promising inhibitor class concerning oncological purposes.

Consequently, it was the goal of the presented study to seek novel hPARP-1 inhibitors by initially using a cell-free, enzyme-based screening assay. An in silico docking study was performed to pre-select potential hPARPi out of the compound library of the Leibniz Institute of Plant Biochemistry (IPB), comprising more than 30,000 natural products from plants and fungi and (semi)synthetic derivatives. Virtually pre-selected potential inhibitor molecules were screened at two concentrations (1 and 10 µM) by using a new variation of a hPARP-1 enzyme assay to identify true hPARP inhibitors. Active compounds were characterized in more detail to evaluate their IC_50_ values of enzyme inhibition. Subsequently, the most active inhibitor candidates were further tested in cell-based assays using different breast cell lines—namely 184B5, MDA-MB-468, MCF-7, HCC1937, and BT-474—to determine its antiproliferative effect in breast cancer cells and its assumed synergistic effect with the DNA-damaging drug temozolomide (TMZ) in comparison with the approved OLP.

## 2. Results

### 2.1. In Silico Pre-Selection of Potential PARPi

**Figure 2:**
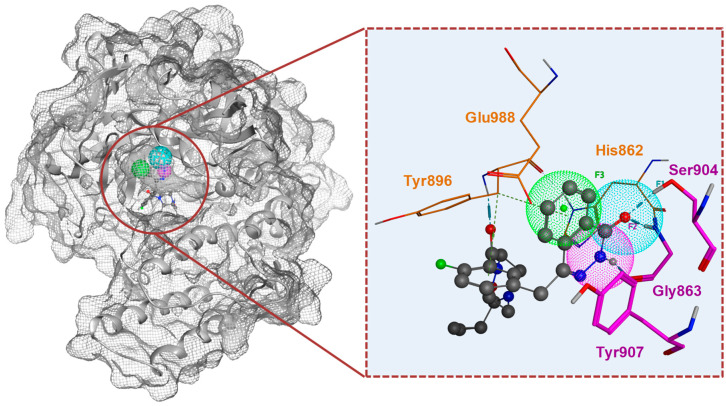
The structure of the ART subdomain of hPARP-1 (PDB code: 3GJW) and the essential pharmacophore features of an established hPARP-1 inhibitor molecule (olaparib, gray balls, and sticks). Position of pharmacophore features are highlighted in the zoom-in. Cyan represents the position of the hydrogen bond acceptor (F1), magenta represents the position of the hydrogen bond donor (F2), and green represents the position of the aromatic ring (F3). Pink-colored amino acid residues represent those that interact directly with the hPARPi and the orange-colored ones represent the blocked H-Y-E triad.

By applying the hPARP ligand to an in silico molecular docking approach based on the established PARPi pharmacophore features (Figure 2), a subset of IPB’s natural and natural-like products library was virtually screened, and compounds were ranked according to the calculated binding energies of their interaction with hPARP-1. Consequently, 49 compounds, which showed the lowest energies upon binding, were selected to be studied in wet lab assays. A further 20 compounds—comprising a benzamide group, i.e., a known hallmark of hPARP-1 inhibitors—were additionally included in the study [33,34]. In summary, 69 compounds (Table S1) were pre-selected and subsequently tested for their in vitro inhibitory potency against hPARP-1. All compounds were synthesized by Dr. Robert Berger and fully characterized by ^1^H NMR, ^13^C NMR, and ESI-MS, with their structures subsequently confirmed [35].

### 2.2. Discovery of Eight Novel PARP Inhibitors

**Figure 3:**
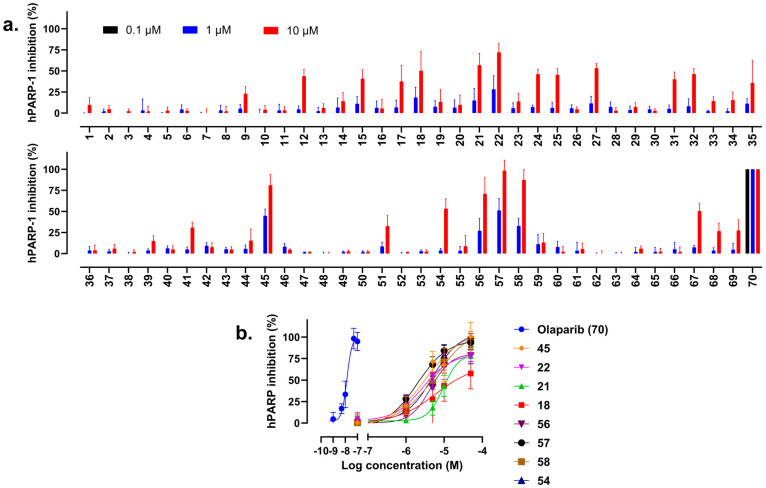
Fast screening to detect the inhibitory impact of the selected compounds on hPARP-1′s catalytic activity using the hPARP-1 enzyme assay. (**a**) Compounds were tested at two different concentrations (1 and 10 µM) to determine the most active hits. OLP (compound **70**) was additionally applied at a concentration of 0.1 µM as a positive control. (**b**) Dose–response curves determining the IC_50_ values of the most active compounds. Results are presented as the mean ± standard deviation calculated based on two biological replicates, each comprising three technical replicates.

**Figure 4:**
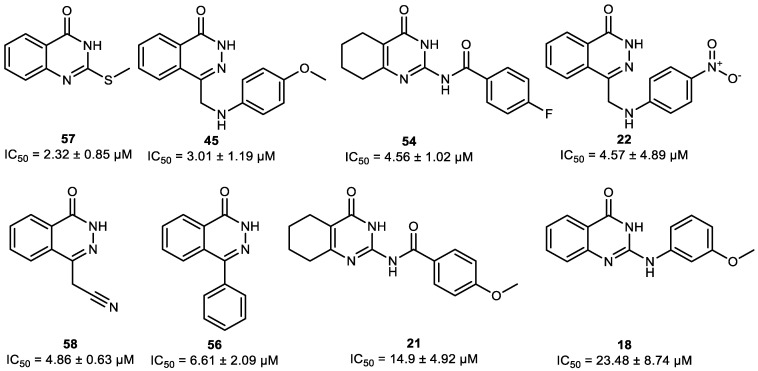
Structures and IC_50_ values of the most active novel hPARPi compounds as determined by using the hPARP-1 enzyme assay.

The 69 pre-selected compounds (Table S1) were assayed for their enzyme inhibition potency by using an established hPARP-1 enzyme assay. Initially, each compound was tested at two concentrations (1 and 10 µM). As summarized in Figure 3a, the compounds **18**, **21**, **22**, **45**, **54**, **56**, **57**, and **58** were found to induce more than 50% inhibition of hPARP-1 when using a concentration of 10 µM. Hence, these compounds were selected to be tested (Figure 3b) at a wider range of concentrations (0, 0.1, 1, 5, 10, and 50 µM), and their IC_50_ values were calculated as shown together with their chemical structures in Figure 4. The approved PARPi drug olaparib (OLP) was tested as a reference. As a result, compound **57** was identified as the most active hPARPi out of the tested compounds, with a calculated IC_50_ value of 2.3 µM. Therefore, compound **57** was selected to be characterized in more detail in further investigations.

### 2.3. mRNA Expression Levels of hPARP-1 and SLFN-11

**Figure 5:**
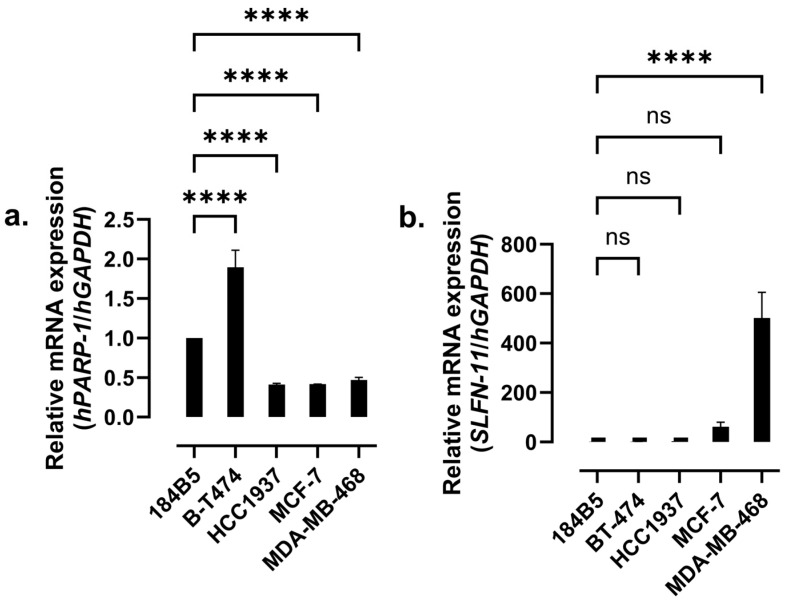
mRNA expression levels of (**a**) *hPARP-1* and (**b**) *SLFN-11*, as determined in the breast cell lines under investigation using RT-qPCR. The expression levels were calculated following the ΔΔCt methodology in relation to the expression of the housekeeping gene *hGAPDH* and were normalized for illustration to the corresponding expression levels in 184B5 healthy breast cells. **** *p*-value < 0.001; ns, not-significant difference.

The mRNA expression levels of hPARP-1 and SLFN-11 in the breast (cancer) cell lines under investigation were measured using RT-qPCR. In contrast to the other cell lines, 184B5 is not a breast cancer cell line, but a healthy chemically transformed epithelial breast cell line. BT-474 cells showed the highest hPARP-1 expression compared to the other cell lines (Figure 5a). However, the expression level of SLFN-11 was found to be significantly higher in MDA-MB-468 compared to the other cell lines, as shown in Figure 5b.

### 2.4. In Vitro Cytotoxicity of Compound ***57***

**Figure 6:**
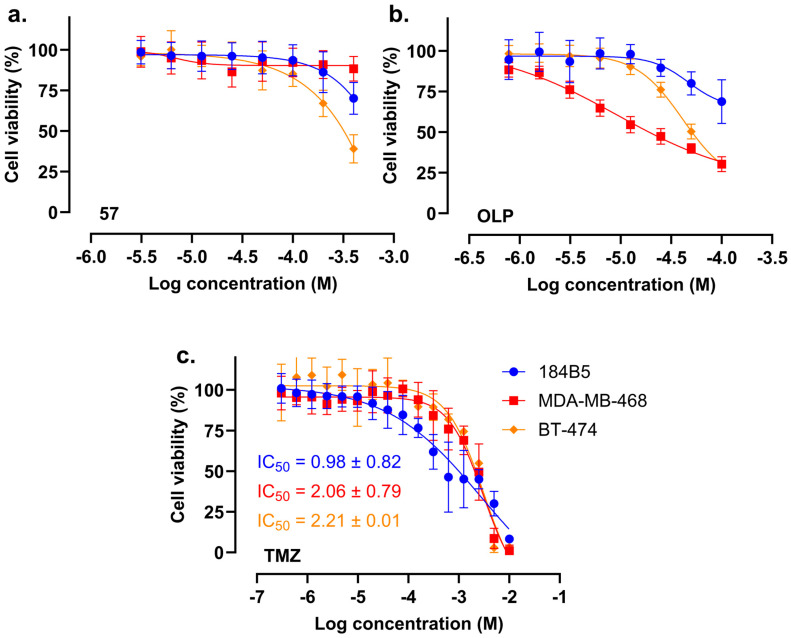
Cell viability dose–response curves of the breast cell lines under investigation upon 72 h treatment with (**a**) compound **57**, (**b**) OLP, and (**c**) TMZ. Cell viability was determined using CV assay. Results are presented as the mean ± standard deviation calculated based on two biological replicates, each comprising three technical replicates.

Compound **57**, as well as olaparib (OLP) and temozolomide (TMZ) as reference compounds, were tested in vitro for their cytotoxic activity against the selected breast (cancer) cell lines by conducting crystal violet (CV) cytotoxicity assays. Each of the compounds were tested in a suitable concentration range, individually adjusted based on their expected activity, as illustrated in Figure 6 and Figure S1. The dose–response data were analyzed using the GraphPad Prism software v10.1, and IC_50_ and IC_10_ values were calculated where possible, based on data using a four parametric non-linear regression analysis. The calculated IC_10_ values of compound **57** in BT-474 cells (100 µM) and of OLP in MDA-MB-468 cells (1.56 µM) were used in subsequent synergistic cytotoxicity assays. The IC_50_ values of TMZ were calculated as well and are illustrated in Table 1 and Table S2.

### 2.5. Compound ***57*** Did Not Exhibit Synergistic Effect in Combination with TMZ

**Figure 7:**
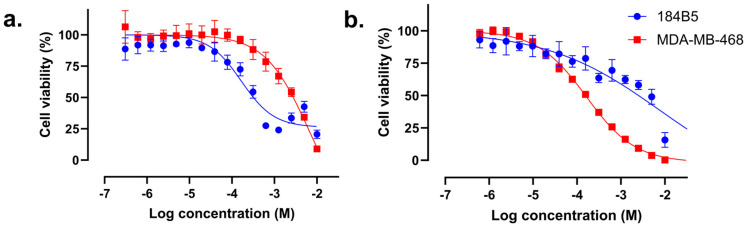
In vitro dose–response curves of 184B5 healthy breast and MDA-MB-468 breast cancer cells. The cells were treated for 72 h with several concentrations of TMZ combined with IC_10_ of (**a**) compound **57** and (**b**) OLP. Cell viability was determined using CV assay. Results are presented as the mean ± standard deviation calculated based on two biological replicates, each comprising three technical replicates.

IC_10_ concentrations of OLP and compound **57** were used as co-treatments with several concentrations of TMZ to explore the possibility of a synergistic enhancement of the cytotoxicity of TMZ. As shown in Table 1 and Table S2, OLP improved the IC_50_ of TMZ against MDA-MB-468 by ≈21-fold, while compound **57** did not induce a synergistic improvement of the TMZ activity (Figure 7 and Figure S2).

### 2.6. Compound ***57*** Does Not Reach the Nucleus-Located hPARP-1 Enzyme

**Figure 8:**
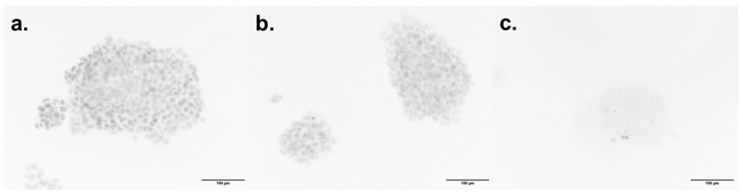
BT-474 cells treated for 72 h with (**a**) complete medium (non-treated control), (**b**) 100 µM of compound **57**, and (**c**) 1.57 µM of OLP, followed by hPARP-1 staining with 250 nM of PARPi-FL for 20 min at 37 °C. The images were captured by using the GFP channel of an EVOS™ FL Auto Imaging System and were processed using FIJI software v2.15. For better signal visibility, the image colors were inverted and are shown here in grey scales, whereby grey represents the detected green fluorescence of the PARPi-FL staining. Scale bar: 100 µm.

With respect to *hPARP-1* expression, the BT-474 cells showed the highest mRNA expression level, as determined by RT-qPCR. Consequently, the BT-474 cell line was chosen for fluorescence microscopic investigations on a supposed spatial co-localization of compound **57** and hPARP-1. The investigation was conducted by using the green, fluorescent PARP ligand PARPi-FL, which occupies the ART domain of the hPARP-1 enzyme and was already proven to be competitively displaced using active PARP inhibitors. Accordingly, PARPi-FL occupied the enzyme pocket of hPARP-1 in the cells’ nuclei and produced green nuclei-located fluorescence, as illustrated in Figure 8a. However, while OLP was able to reach the hPARP-1 enzymes in the nuclei, competing with and replacing PARPi-FL from binding to the enzyme and, hence, reducing the nuclear PARPi-FL fluorescence (as shown in Figure 8b), compound **57** obviously failed to reach the nuclei of BT-474 cells (Figure 8c).

## 3. Discussion

**Figure 9:**
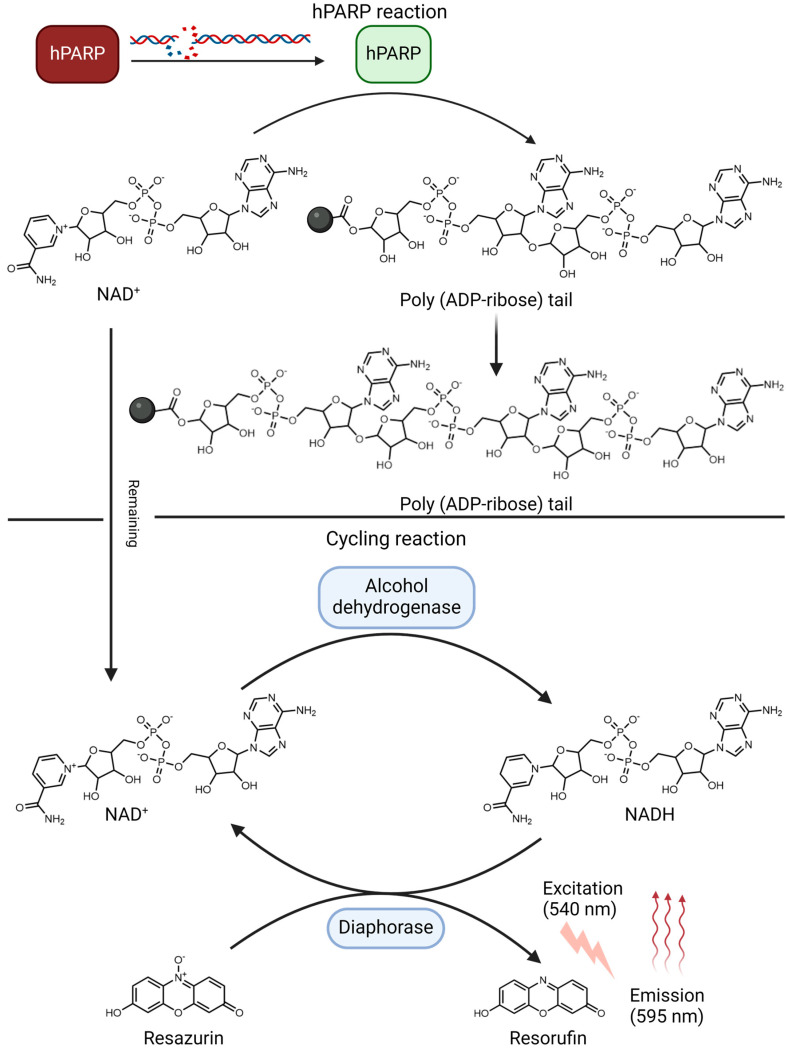
The concept of the modified hPARP-1 enzyme assay. The assay version presented in this paper consists of a hPARP enzyme reaction and a cycling reaction. In the hPARP reaction, the hPARP enzyme is activated in the presence of activated, i.e., strand breaks-containing DNA and uses the NAD^+^ substrate to add poly(ADP-ribose) tails as posttranslational modification to itself. In the second step, the cycling reaction allows a catalytically amplified fluorescent read-out of the remaining NAD^+^ substrate that, in case of successful hPARPi treatment, is not used up when hPARP is inhibited. NAD^+^ is transformed to NADH+H^+^, which is used to reduce resazurin to resorufin (excitation/emission: 540/595) the fluorescence of which serves as final assay read-out and indirectly indicates the activity of the hPARP enzyme. The scheme was created with BioRender.com.

By conducting a pharmacophore-based in silico screening of the Leibniz Institute of Plant Biochemistry’s (IPB) compound library, 69 compounds were defined as potential hPARP inhibitors (hPARPi). Thus, a new hPARP-1 enzyme assay variant was implemented to prove these pre-selected compounds for actual hPARP inhibition. The hPARP-1 assay was conducted based on the fact that the hPARP-1 enzyme requires NAD^+^ as a cofactor and a supply of ADP-ribose units. Upon activation of hPARP-1 in the presence of activated DNA, i.e., DNA containing strand breaks, the enzyme starts consuming NAD^+^. However, the inhibition of hPARP-1 leads to a reduction of the NAD^+^ consumption, which can be quantified using a cycling reaction (Figure 9). The cycling reaction utilizes the remaining NAD^+^, i.e. NAD^+^ that was not used up by the hPARP-1 reaction, to reduce resazurin to its fluorescent derivative resorufin. Moreover, the fluorescent resorufin signal gets amplified due to the redundant cyclic catalysis based on the combination of diaphorase and alcohol dehydrogenase. In brief, the amount of the remaining NAD^+^ is directly proportional to the inhibition of the hPARP-1 enzyme using a hPARPi. Initially, the linearity of the cycling reaction that detects the remaining NAD^+^ concentration available in the reaction mixture was confirmed. Several concentrations of NAD^+^ were measured and the obtained absolute fluorescent values were analyzed in a calibration curve. Afterwards, several concentrations of hPARP-1 were tested to determine which enzyme concentration, i.e., enzyme activity as defined by a unit count, was suitable to induce around 80% consumption of 50 nM of NAD^+^, which was found to be 2 U of the used hPARP-1 batch. However, the optimal hPARP-1 concentration to be used should be determined for each enzyme batch individually due to its high dependency on each enzyme preparation and its specific enzymatic activity.

Initially, as experimental proof of the assay reliability, the aforementioned hPARP-1 enzyme assay protocol was used to determine the IC_50_ value of the reference PARPi OLP. A value of 13 nM was in good agreement with the 5 nM IC_50_ value previously published in the literature on the use of OLP against hPARP-1 [36]. Consequently, the enzyme assay was used to screen our in silico pre-selected compounds for hPARP-1 inhibition. Compounds **18**, **21**, **22**, **45**, **54**, **56**, **57**, and **58** (shown in Figure 4) inhibited hPARP-1 and were further tested to determine their IC_50_ values, which ranged from 2.3 to 23.5 µM.

**Figure 10:**
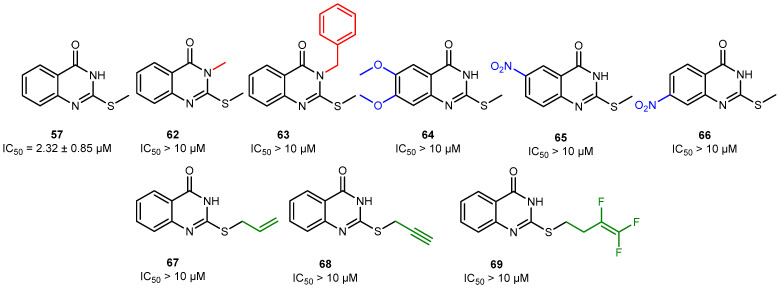
Structure–activity relationship (SAR) considerations regarding the scaffold family of compound **57** (IC_50_ = 2.3 µM) representing the most active hPARPi compound newly identified. The IC_50_ values of **62**–**69** were higher than 10 µM. Color codes refer to SAR discussions in the main text.

**Figure 11:**
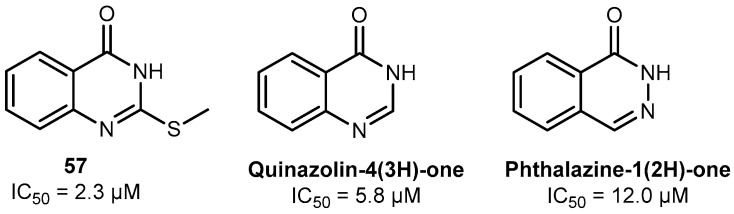
Structures of compound **57**, quinazolin-4(*3H*)-one, and phthalazin-1(*2H*)-one (the OLP core structure).

**Figure 12:**
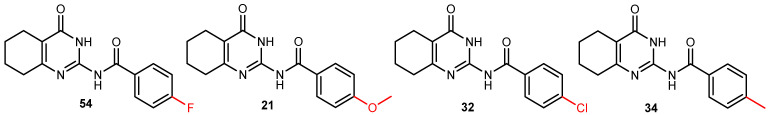
Structure–activity relationship (SAR) considerations regarding the scaffold family of compound **54**.

Compound **57** (2-(methylthio)quinazolin-4(*3H*)-one) was found to be the most active hPARPi, with an IC_50_ of 2.3 µM against hPARP-1. Accordingly, the binding of compound **57** to the ART subdomain of hPARP-1 was studied by in silico molecular modeling and it was found exhibiting the interactions that were previously claimed to be essential for the activity of hPARPi, as shown in Figure S3 (cf. also Figure 2). These are the hydrophobic interactions with Tyr907 and H-bonds with Ser904 and Gly863. Concerning the structure–activity relationship (SAR, cf. Figure 10, Figure 11 and Figure 12), several features were identified to be essential for its activity as a hPARPi. By comparing compound **57** with compounds **62** and **63**, it was noticed that any substitution at the nitrogen position 3 (Figure 10; red part of the structures) led to a complete abolishment of hPARP-1 inhibitor activity. The rationale behind this observation is that the replaced hydrogen atom seems to be essential for the formation of an H-bond with the oxygen group of the glycine at position 863 of hPARP-1, as described in the literature [37]. Moreover, the modifications at meta or para positions in the compounds **64**, **65**, and **66** (Figure 10; blue parts of the structures) lead to a dramatic decrease in activity as it disturbs the crucial hydrophobic interaction that has to be formed with the tyrosine residue of the ART subdomain [28]. A modification at the thiomethyl group of compound **57**, as realized in compounds **67**, **68,** and **69** (Figure 10, green part of the structures), does not lead to complete loss of the compounds’ activity; however, it causes a reduction in the PARPi activity by at least four-fold. Compound **57** (IC_50_ = 2.3 µM) also showed a 2.5-fold higher hPARP-1-inhibiting activity compared to its core structure quinazolin-4(*3H*)-one shown in Figure 11 (IC_50_ = 5.8 µM), lacking the thiomethyl group [38,39].

Besides the compound **57** scaffold, another new core structure (4-methyl-*N*-(4-oxo-3,4,5,6,7,8-hexahydroquinazolin-2-yl)benzamide) was identified to exhibit hPARP inhibitory activity. Based on the available compounds, several modifications were identified that strongly affect its activity. The most active compound sharing that core structure was compound **54** (IC_50_ = 4.56 µM, see Figure 12) with a fluoride group at the para position of the phenyl group. However, the replacement of the fluoride group by either the methoxy, methyl, or chloride group, as realized in the compounds **21**, **32**, and **34** (Figure 12; red part of the structures) causes a decline in the compounds’ PARPi activity by at least three-fold. The high activity of this family of compounds could be related to the presence of an additional benzamide group attached to the core structure of the aforementioned compound **57** family in which the aromatic group may form a π–π interaction with Tyr896 similar to that formed by the benzyl group in the case of OLP as shown in Figure 2 [40].

According to several previous studies, the cancer cells’ susceptibility to hPARP inhibition should be inversely proportional to their BRCA gene expression [24,41]. Nevertheless, during the last years, various studies highlighted a new, more important cornerstone for the prediction of hPARPi susceptibility, which was the expression of the *SLFN-11* gene [26,42,43]. Hence, the expression levels of *SLFN-11* in the breast (cancer) cell lines under investigation were tested by using RT-qPCR, along with those of *hPARP-1* itself. The highest *SLFN-11* expression level was found in MDA-MB-468 triple-negative breast cancer (TNBC) cells, which was more than 10-fold higher compared to MCF-7 hormone receptor-positive (HR+) breast cancer cells and at least 300-fold higher compared to the other breast (cancer) cell lines. Based on the determined *SLFN-11* expression levels in conjunction with the given *hPARP-1* expression, it was expected to observe the highest impact of the PARPi reference drug OLP as well as the newly discovered PARPi compounds, in particular of compound **57**, in MDA-MB-468 cells. Indeed, OLP improved the cytotoxicity of TMZ towards MDA-MB-468 by approximately 21-fold, in which TMZ induces the formation of single-strand breaks, while OLP inhibits the repair of the DNA, both together leading to enhanced cellular toxicity and cell death. Additionally, OLP as a monotherapy also showed good and selective cytotoxic activity against MDA-MB-468. However, such an effect was not observed with compound **57**, despite its potent hPARPi activity. One possible explanation for that result could be the too weak cellular and/or nuclear permeability for compound **57**, and therefore its failure to reach the hPARP-1 enzymes in the nuclei [44]. Hence, to investigate compound **57′s** capacity to penetrate the cellular membranes, fluorescence microscopy was performed. For that purpose, BT-474 cells, showing the highest expression of *hPARP-1* (see Figure 5), were stained with PARPi-FL. That green, fluorescent PARP ligand was developed as an imaging tool for binding at the NAD^+^ binding pocket of PARP enzymes. Consequently, PARPi-FL can be used to screen for hPARP inhibitors competitively displacing the ligand from its binding to the hPARP-1 enzyme. By comparing the images shown in Figure 8, OLP led to complete PARPi-FL displacement and dissolution of its fluorescent signal, indicating high affinity binding to the hPARP-1 enzymes located in the cells’ nuclei. In contrast, compound **57** did not impact the fluorescent appearance of the stain, indicating a lack of hPARP-1 enzyme binding in the cell’s nucleus. This could be an indication of its poor nuclear and/or cellular penetration.

Nonetheless, compound **57** was determined with a five-fold higher PARPi activity at the enzyme level compared to the core structure of OLP, phthalazin-1(*2H*)-one, (IC_50_ = 12 µM), as illustrated in Figure 11 [45], which indicates that the new compound **57** scaffold, nevertheless, could be very promising for the advancement of PARPi development, if bio-availability can be improved.

The first steps of a hit-to-lead strategy for the determination of novel hPARP-1 inhibitors have been taken in this study. Starting with a target validation as shown by the confirmation of the impact of the clinically proven hPARP inhibitor (OLP) against the hPARP-1 enzyme and PARP inhibitor-sensitive cell lines. The second step was the implementation of an hPARP-1 enzyme assay that is easy, robust, cheap, and up-scalable to perform a high-throughput screening for hPARP-1 inhibitors. It was applied to determine several hit compounds which indicate the first step of a lead compound generation. These compounds can be further improved through lead optimization as previously performed with phthalazin-1(*2H*)-one leading to the development of OLP. Initially, for the OLP mother compound phthalazin-1(*2H*)-one, relatively weak PARP inhibitor activity in cell-based assays was also reported. However, due to its promising prospect, step-by-step molecule modifications and optimizations towards the very potent PARPi OLP were implemented. The structural modification by the introduction of a benzyl group at position 4 led to the remarkable improvement of the compound’s IC_50_ from 12 µM to 550 nM and led to an also cellularly active hPARP inhibitor [46]. By introducing a fluoride group at the para position of the benzyl group, a further improvement of the permeability and potency was gained. Further introduction of the piperazine group at the meta position of the benzyl group improved the solubility of the compound [47,48]. Modifications, like those illustrated for OLP, might be used as well for further lead optimization of the newly discovered scaffold **57**, which could unveil novel molecules with even higher activity compared to OLP, as indicated by the lower IC_50_ of core **57** compared to phthalazine-1(*2H*)-one, the OLP core structure, as shown in Figure 11. Such optimized derivatives could then be applied in cancer therapy as potential adjuvants in combination with DNA damaging agents, especially to treat those tumors characterized by overexpression of *SLFN-11* making them more sensitive to DNA damage as previously proven by several studies and also shown in this study by the potentiation of the cytotoxic effect of temozolomide (TMZ) towards the highly SLFN-11 expressing cell line MDA-MB-468 upon addition of the bioavailable hPARP-1 inhibitor olaparib (OLP).

## 4. Materials and Methods

### 4.1. Chemicals and Cell Lines

The synthesis route and analytical data of compound **57**, found to be most active one in this study, are exemplarily described in the Supplementary Information (Scheme S1). Further syntheses routes of other derivatives tested were published recently [34].

Deoxyribonucleic acid sodium salt from calf thymus, MgCl_2_, NAD^+^, resazurin, alcohol dehydrogenase, diaphorase, and crystal violet were purchased from Sigma-Aldrich (St. Louis, MO, USA). High glucose DMEM with glutamine, 0.05% trypsin-EDTA, FCS, RPMI 1640, PBS, glutamine, and penicillin/streptomycin were bought from Capricorn Scientific (Ebsdorfergrund, Germany); the Endopan 3 kit was from PAN-Biotech (Aidenbach, Germany). TRIS, agarose, and paraformaldehyde were supplied by Carl Roth (Karlsruhe, Germany). Recombinant hPARP-1 and PARPi-FL were purchased from Bio-Techne (Minneapolis, MN, USA), DMSO from Duchefa Biochemie (Haarlem, The Netherlands), olaparib from Hycultec GmbH (Beutelsbach, Germany), and temozolomide from MedChemExpress (Monmouth Junction, NJ, USA). The qPCR primers were synthesized and supplied by Eurofins Genomics (Ebersberg, Germany). The RNA miniprep kit was purchased from Zymo Research (Freiburg, Germany), qPCR GreenMaster from Jena Bioscience (Jena, Germany), and RevertAid RT Reverse Transcription Kit from Thermo Fisher Scientific (Waltham, MA, USA).

Several breast cell lines representing diverse types of breast cancer were investigated in the study. HCC1937 and MDA-MB-468 represent triple-negative breast cancer (TNBC), MCF-7 as a hormone receptor-positive (HR+) cell line, and BT-474 as *HER2/neu*-positive (HER2+) cell line [49]. Moreover, the 184B5 cell line is chemically transformed; it originates from normal mammary tissue representing healthy breast cells [50]. All cell lines were obtained from the cell lines stock of the Leibniz Institute of Plant Biochemistry (IPB; Halle (Saale), Germany) and were originally purchased from DSMZ (Braunschweig, Germany) and ATCC (Manassas, VA, USA). 184B5 was cultured using the Endopan 3 kit, while MDA-MB-468 was cultured in high glucose DMEM supplemented with 10% (*v*/*v*) heat-inactivated FCS. BT-474, HCC1937, and MCF-7 were cultured in RPMI 1640 medium supplemented with 10% (*v*/*v*) heat-inactivated FCS, 1% (*v*/*v*) L-glutamine, and 1% (*v*/*v*) penicillin/streptomycin. Cells were grown in their media in a humidified atmosphere with 5% CO_2_ at 37 °C. The confluence was monitored daily using light microscopy. Whenever the confluency reached 90%, cells were detached using 0.05% trypsin-EDTA and diluted as indicated by the American Type Culture Collection (ATCC) guidelines. The cell density used for seeding depended on the surface area of the plates used. For 96-well plates and 6-well plates, 6 × 10^3^ cells/well and 1.5 × 10^5^ cells/well, respectively, were used.

### 4.2. In Silico PARP-PARPi Molecular Docking

To screen the compound library at the Leibniz Institute of Plant Biochemistry, comprising approximately 25,000 compounds (15% natural products, 13% semisynthetic, and the remainder synthetic), a pharmacophore-based in silico docking study was conducted to pre-select potential hPARP-1 inhibitors. The chemoinformatic investigation was conducted by using the molecular modeling software package MOE version 2022.2 (Molecular Operating Environment (MOE) 2022.2; Chemical Computing Group ULC, Montreal, QC, Canada). The X-ray structure of the hPARP-1 enzyme was obtained from the protein databank (PDB code: 3GJW) [51]. The protein structure was initially prepared for the docking simulations by adding the missing hydrogens using the 3D protonation feature of MOE. Afterwards, a pharmacophore was designed based on interactions with the enzyme’s ART domain that were found to be essential for the inhibitory effect of established hPARP-1 inhibitors, namely olaparib and rucaparib. Three interactions with the ART domain were found crucial for the inhibitor binding, namely (i) the hydrophobic interaction between an aromatic ring of the inhibitor (green, Figure 2) and Tyr907 and Try896, (ii) the presence of a hydrogen bond acceptor (cyan, Figure 2) to form a H-bond with the Ser904, and (iii) a hydrogen bond donor (magenta, Figure 2) which forms a H-bond with the Gly863. These interactions were found to block the ART subdomain and prevent the interaction of the NAD^+^ with its binding H-Y-E triad. For each compound, 5 out of 100 poses were generated using MOE. Subsequently, the created pharmacophore was used to dock the compounds of IPB’s library. The binding energies were calculated using MOE software with the forcefield-based scoring function GBVI/WSA ΔG for each compound pose and used to rank the compounds with respect to their proposed potential to inhibit the hPARP-1 enzyme. The top compounds (69 compounds in total) were visually inspected and selected for further experiments.

### 4.3. hPARP-1 Enzymatic Assay (New Variation)

The putative PARP inhibitors, which were pre-selected in the in silico study, were subsequently evaluated by using an optimized biochemical hPARP-1 enzyme assay. The experiment was performed in black half-area 96-well plates. For the PARP reaction, 12.5 µL of PARP reaction solution were added. The solution was composed of 0.5 µg deoxyribonucleic acid (DNA) sodium salt from calf thymus, 2 U of recombinant hPARP-1, and 1 µL of the compound of interest in PARP reaction buffer (50 mM TRIS and 2 mM MgCl_2_ in ddH_2_O, pH = 8) [52]. These test compounds’ working solutions were prepared from 20 mM DMSO stock solutions and diluted using PARP reaction buffer to reach 25× of the required final concentration to be tested. As a positive control, 1 µL of 2.5 µM OLP was used (final concentration: 100 nM), and as a negative control, 1 µL of the PARP reaction buffer instead of the test item’s working solution. Afterwards, 12.5 µL of NAD^+^ solution were added to all wells to reach a final NAD^+^ concentration of 50 nM, and to start the enzyme reaction.

Besides PARP reaction wells, NAD^+^ calibration wells were included to measure the linearity of the detection of NAD^+^ concentrations. In each well, 12.5 µL of NAD^+^ calibration solution were added. The solution was composed of 0.5 µg deoxyribonucleic acid (DNA) sodium salt from calf thymus in PARP reaction buffer. Finally, 12.5 µL of decreasing concentrations of NAD^+^ solution were added to the calibration wells (final concentrations: 50, 40, 30, 20, 10, 0 nM).

Subsequently, the assay plate including both the PARP reaction and NAD^+^ calibration wells was gently shaken and incubated at 25 °C in the dark for 90 min. After incubation, 25 µL of detection solution were added to each well. The detection solution was composed of 50 µM resazurin, 2.1% (*v*/*v*) ethanol, 2 U alcohol dehydrogenase, and 0.2 U diaphorase in PARP reaction buffer [53]. The assay plate was again gently shaken and incubated at 25 °C in the dark for 40 min. Finally, after reductive conversion of resazurin to resorufin, fluorescence was measured at excitation/emission wavelengths of 545/595 nm using a SpectraMax iD5 plate reader (Molecular Devices, San Jose, CA, USA). The obtained data were expressed as mean values normalized by using the positive control as 100% inhibition and negative control as 0% inhibition.

Following the aforementioned protocol, the compounds that were predicted by the in silico study as putative hPARP-1 inhibitors were tested at two different concentrations (1 and 10 µM). Those that were found to induce a significant reduction in hPARP-1 activity were further tested at five more concentrations (50, 10, 5, 1, 0.1 µM) to determine their IC_50_ values against hPARP-1 enzyme activity. The IC_50_s were calculated by using GraphPad Prism software and a non-linear four-parametric regression function.

### 4.4. Differential Gene Expression

The gene expression levels of *hPARP-1* and *SLFN-11*, a predictive biomarker for hPARP sensitivity of cancer cells [22,23], in the breast cell lines under investigation were quantified by using RT-qPCR. For that purpose, RNA was isolated from 5 × 10^6^ cells of each cell line using an RNA miniprep kit. Afterward, the integrity and concentration of the isolated RNA were determined by using 1% (*w*/*v*) agarose gel and a SpectraMax iD5 with SpectraDrop^TM^ Micro-Volume kit (Molecular Devices, San Jose, CA, USA). A measure of 500 ng of isolated RNA was utilized to produce first-strand cDNA by using a RevertAid RT Reverse Transcription kit. Finally, 12.5 ng of the synthesized cDNA was used as a template for the RT-qPCR reaction. The qPCR primers were designed using the open-source Primer Design Tool from NCBI (Table S3). qPCR GreenMaster mix was used as a reporter for the amplicon amplification which was performed by using a CFX96^TM^ Real-Time PCR Detection System (Bio-Rad, Hercules, CA, USA). Finally, the data were analyzed according to the ΔΔCt methodology and normalized to the expression of the housekeeping gene glyceraldehyde 3-phosphate dehydrogenase (*hGAPDH*) [54]. RNA isolation, cDNA synthesis, and the qPCR were performed as indicated by the manufacturers’ guidelines of the used kits [55].

### 4.5. In Vitro Cell Viability Assay

The impact of the putative PARP-1 inhibitors on the breast (cancer) cells’ viability was determined by using the crystal violet (CV) assay, an assay which quantifies the number of adherent cells and hence indirectly indicates the number of viable cells since cell adherence is a characteristic of these types of cell lines as long as they are viable; upon death, cells lose their adherence ability [56]. Cells were seeded in 96-well cell culture plates and were allowed to adhere for 24 h in a humidified atmosphere with 5% CO_2_ at 37 °C. Afterward, cells were treated with the test items at several concentrations for 72 h. Complete medium and 125 µM of digitonin (a strongly cytotoxic saponin) in medium, were used as negative and positive control, respectively [57]. After finalization of the intended 72 h treatment, the treatment solutions were discarded, and the cells were washed once with PBS. Subsequently, the cells were fixed by incubation in 4% (*v*/*v*) paraformaldehyde in PBS for 15 min at room temperature (RT). The fixing solution was discarded, and the cells were allowed to dry at RT for 15 min. The cells were stained with 0.1% (*w*/*v*) crystal violet in PBS for 15 min at RT, then washed with ddH_2_O and dried overnight at RT. Finally, the stain was dissolved using 33% (*v*/*v*) acetic acid, and the CV absorbance at 570 nm and at the reference wavelength of 670 nm were measured using a SpectraMax iD5 plate reader [58]. The obtained absorbance data were normalized based on the negative and positive controls representing 100% and 0% cell viability, respectively. IC_50_ values were calculated by using a four-parametric non-linear regression analysis in GraphPad Prism software [59].

### 4.6. In Vitro Synergistic Cell Viability Assay

To investigate whether the most promising compound, **57**, could act synergistically in conjunction with an established anti-cancer drug, it was tested in combination with temozolomide (TMZ). For that purpose, cells were seeded in 96-well plates and incubated in a humidified atmosphere with 5% CO_2_ for 24 h at 37 °C. Afterward, the medium was discarded, and cells were treated for 72 h with 100 µM of compound **57** or 1.56 µM of olaparib (OLP), combined with several concentrations of TMZ in 100 µL of the medium. Finally, cell viability was determined by using the CV assay, and IC_50_ values were calculated as mentioned above [60].

### 4.7. Fluorescence Microscopy

For the determination of the interaction of the proposed PARPi and the PARP binding pocket inside the treated cells, a fluorescence microscopic inspection was performed. Using a green fluorescently labeled molecule named PARPi-FL that is characterized by the ability to occupy specifically the binding pocket of the PARP enzyme. Moreover, in case of its competitive replacement by PARPi molecules a reduction in its fluorescent signal is detectable. Thus, cells were seeded in 6-well plates and incubated in a humidified atmosphere with 5% CO_2_ for 24 h at 37 °C. After incubation, cells were treated with either compound **57** at 100 µM or OLP at 1.56 µM for 72 h under standard growth conditions. Subsequently, the treatment solutions were discarded, and the cells were washed with PBS. Afterward, the cells were stained by using 250 nM of PARPi-FL in PBS for 20 min. Finally, cells were imaged by using the GFP channel of an EVOS™ FL Auto Imaging System (Thermo Fisher Scientific, Waltham, MA, USA) [61,62].

## 5. Conclusions

This study aimed to implement a cost-effective screening methodology to discover novel hPARP inhibitors. Therefore, a cell-free hPARP-1 enzyme assay was established, optimized, and utilized to screen for hPARP-1 inhibitors from the compound library of the Leibniz Institute of Plant Biochemistry (IPB, Halle (Saale), Germany). Out of 69 in silico pre-selected compounds, 6 molecules were detected to inhibit the hPARP-1 enzyme at exceptionally low micromolar concentrations. The most active candidate was compound **57,** with an IC_50_ of 2.3 µM. However, the compound failed to have impact in cell-based assays, seemingly due to its weak nuclear membrane penetration. Nevertheless, most interestingly, the lead molecule, **57**, showed five-fold higher PARPi activity than the olaparib (OLP) core structure (phthalazin-1(*2H*)-one) itself. Therefore, further chemical substitution and optimization based on compound **57** as a core structure can be expected to lead to a novel PARP inhibitor class with PARPi activities and drug likeliness in the same activity range or better than OLP and other approved PARP inhibitors. Moreover, besides compound **57**, a second new and promising core structure for novel PARP inhibitors was unveiled. Even though compound **54** showed lower activity compared to compound **57**, its PARPi activity was still two-fold higher compared to the OLP core structure. Furthermore, a first structure–activity relationship (SAR) was devised, and several features were highlighted that affect the interaction of the PARPi molecules with the hPARP-1 enzyme, consequently determining their inhibitory potency. These findings will help to improve the development and optimization of novel hPARP inhibitors.

## Figures and Tables

**Table 1:** Summary of the calculated IC_50_ values (given in mM) of TMZ, TMZ combined with 100 µM of compound **57**, and TMZ combined with 1.56 µM of OLP, as tested against the breast cell lines. Cells were treated for 72 h and assayed using the CV assay.

Treatment\IC_50_ [mM]	184B5	MDA-MB-468
TMZ	0.98 ± 0.82	2.06 ± 0.79
TMZ + compound **57**	0.32 ± 0.07	2.77 ± 0.25
TMZ + OLP	4.95 ± 3.90	0.13 ± 0.04

## Data Availability

All data underlying the analyses and findings of this study are available from the corresponding author upon reasonable request.

## Supplementary Materials

The following supporting information can be downloaded at: https://www.mdpi.com/article/10.3390/molecules30132728/s1, Figure S1. Cell viability dose–response curves of the breast cell lines under investigation upon 72 h treatment with **a** compound **57**, **b** OLP, and **c** TMZ. Cell viability was determined by using crystal violet (CV) assay. Results are presented as the mean ± standard deviation. The mean is calculated from two biological replicates, each comprising three technical replicates. Figure S2. Dose-dependent curves of several breast cell lines. The cell lines were treated with several concentrations of TMZ combined with IC_10_ of **a** compound **57** and **b** OLP for 72 h, and the viability was determined using CV assay. Results are presented as the mean ± standard deviation. The mean is calculated from two biological replicates, each comprising three technical replicates. Figure S3. The structure of the ART subdomain of hPARP-1 (PDB code: 3GJW) interacting with the newly identified hPARP inhibitor compound **57** (cyan). Closeup view of the active site shows the hydrogen bond interactions with Ser904 and Gly863, and π–π stacking interactions with Try907. Figure S4. The structure of the ART subdomain of hPARP-1 active site with the overlayed PARP inhibitor compound **57** (cyan), Quinazolin-4(*3H*)-one (orange), and Phthalazin-1(*2H*)-one (pink). Scheme S1. Synthesis and analytical data of compound **57**. Table S1. List of the compounds identified as potential hPARP-1 inhibitors by the in silico hPARP-1 docking and screening study. Table S2. Summary of the calculated IC_50_ values (given in mM) of TMZ, TMZ combined with 100 µM of compound **57**, and TMZ combined with 1.56 µM of OLP, as tested against the breast cell lines. Cells were treated for 72 h, and cell viability was determined by using the CV assay. Table S3. Sequences of the qPCR primers used in the gene expression study. Table S4. Binding energies of compound **57**, Olaparib, and their underlying core structures interacting with the ART domain of hPARP-1 from molecular docking using MOE 2022.2 (Molecular Operating Environment (MOE), 2022.02 Chemical Computing Group ULC, Montreal, QC, Canada) software.

## References

[1] Bray F., Laversanne M., Sung H., Ferlay J., Siegel R.L., Soerjomataram I., Jemal A. (2024). Global cancer statistics 2022: GLOBOCAN estimates of incidence and mortality worldwide for 36 cancers in 185 countries. CA A Cancer J. Clin..

[2] Weigelt B., Geyer F.C., Reis-Filho J.S. (2010). Histological types of breast cancer: How special are they?. Mol. Oncol..

[3] Briest S., Stearns V. (2009). Tamoxifen metabolism and its effect on endocrine treatment of breast cancer. Clin. Adv. Hematol. Oncol. HO.

[4] Morgan M.M., Arendt L.M., Alarid E.T., Beebe D.J., Johnson B.P. (2019). Mammary adipose stromal cells derived from obese women reduce sensitivity to the aromatase inhibitor anastrazole in an organotypic breast model. FASEB J..

[5] Baker J.H.E., Kyle A.H., Reinsberg S.A., Moosvi F., Patrick H.M., Cran J., Saatchi K., Häfeli U., Minchinton A.I. (2018). Heterogeneous distribution of trastuzumab in HER2-positive xenografts and metastases: Role of the tumor microenvironment. Clin. Exp. Metastasis.

[6] Cortés J., Kim S.-B., Chung W.-P., Im S.-A., Park Y.H., Hegg R., Kim M.H., Tseng L.-M., Petry V., Chung C.-F. (2022). Trastuzumab Deruxtecan versus Trastuzumab Emtansine for Breast Cancer. New Engl. J. Med..

[7] Tečić Vuger A., Šeparović R., Vazdar L., Pavlović M., Lepetić P., Šitić S., Bajić Ž., Šarčević B., Vrbanec D. (2020). Characteristics and Prognosis of Triple-Negative Breast Cancer Patients: A Croatian Single Institution Retrospective Cohort Study. Acta Clin. Croat..

[8] Gupta G.K., Collier A.L., Lee D., Hoefer R.A., Zheleva V., Siewertsz van Reesema L.L., Tang-Tan A.M., Guye M.L., Chang D.Z., Winston J.S. (2020). Perspectives on Triple-Negative Breast Cancer: Current Treatment Strategies, Unmet Needs, and Potential Targets for Future Therapies. Cancers.

[9] Rissel D., Heym P.P., Thor K., Brandt W., Wessjohann L.A., Peiter E. (2017). No silver bullet—Canonical Poly(ADP-Ribose) Polymerases (PARPs) are no universal factors of abiotic and biotic stress resistance of Arabidopsis thaliana. Front. Plant Sci..

[10] Lüscher B., Bütepage M., Eckei L., Krieg S., Verheugd P., Shilton B.H. (2018). ADP-Ribosylation, a Multifaceted Posttranslational Modification Involved in the Control of Cell Physiology in Health and Disease. Chem. Rev..

[11] Matta E., Kiribayeva A., Khassenov B., Matkarimov B.T., Ishchenko A.A. (2020). Insight into DNA substrate specificity of PARP1-catalysed DNA poly(ADP-ribosyl)ation. Sci. Rep..

[12] van Beek L., McClay É., Patel S., Schimpl M., Spagnolo L., Maia de Oliveira T. (2021). PARP Power: A Structural Perspective on PARP1, PARP2, and PARP3 in DNA Damage Repair and Nucleosome Remodelling. Int. J. Mol. Sci..

[13] Ali A.A.E., Timinszky G., Arribas-Bosacoma R., Kozlowski M., Hassa P.O., Hassler M., Ladurner A.G., Pearl L.H., Oliver A.W. (2012). The zinc-finger domains of PARP1 cooperate to recognize DNA strand breaks. Nat. Struct. Mol. Biol..

[14] Alemasova E.E., Lavrik O.I. (2019). Poly(ADP-ribosyl)ation by PARP1: Reaction mechanism and regulatory proteins. Nucleic Acids Res..

[15] Herceg Z., Wang Z.-Q. (2001). Functions of poly (ADP-ribose) polymerase (PARP) in DNA repair, genomic integrity and cell death. Mutat. Res./Fundam. Mol. Mech. Mutagen..

[16] Loeffler P.A., Cuneo M.J., Mueller G.A., DeRose E.F., Gabel S.A., London R.E. (2011). Structural studies of the PARP-1 BRCT domain. BMC Struct. Biol..

[17] Cuneo M.J., Gabel S.A., Krahn J.M., Ricker M.A., London R.E. (2011). The structural basis for partitioning of the XRCC1/DNA ligase III-α BRCT-mediated dimer complexes. Nucleic Acids Res..

[18] Beniey M., Haque T., Hassan S. (2019). Translating the role of PARP inhibitors in triple-negative breast cancer. Oncoscience.

[19] Spigel D.R. (2012). PARP Inhibitors in Lung Cancer. J. Thorac. Oncol..

[20] Higuchi F., Nagashima H., Ning J., Koerner M.V.A., Wakimoto H., Cahill D.P. (2020). Restoration of Temozolomide Sensitivity by PARP Inhibitors in Mismatch Repair Deficient Glioblastoma is Independent of Base Excision Repair. Clin. Cancer Res..

[21] Farago A.F., Yeap B.Y., Stanzione M., Hung Y.P., Heist R.S., Marcoux J.P., Zhong J., Rangachari D., Barbie D.A., Phat S. (2019). Combination Olaparib and Temozolomide in Relapsed Small-Cell Lung Cancer. Cancer Discov..

[22] Papadimitriou M., Mountzios G., Papadimitriou C.A. (2018). The role of PARP inhibition in triple-negative breast cancer: Unraveling the wide spectrum of synthetic lethality. Cancer Treat. Rev..

[23] Zhu Y., Liu Y., Zhang C., Chu J., Wu Y., Li Y., Liu J., Li Q., Li S., Shi Q. (2018). Tamoxifen-resistant breast cancer cells are resistant to DNA-damaging chemotherapy because of upregulated BARD1 and BRCA1. Nat. Commun..

[24] Farmer H., McCabe N., Lord C.J., Tutt A.N.J., Johnson D.A., Richardson T.B., Santarosa M., Dillon K.J., Hickson I., Knights C. (2005). Targeting the DNA repair defect in BRCA mutant cells as a therapeutic strategy. Nature.

[25] Zoppoli G., Regairaz M., Leo E., Reinhold W.C., Varma S., Ballestrero A., Doroshow J.H., Pommier Y. (2012). Putative DNA/RNA helicase Schlafen-11 (SLFN11) sensitizes cancer cells to DNA-damaging agents. Proc. Natl. Acad. Sci. USA.

[26] Coleman N., Zhang B., Byers L.A., Yap T.A. (2021). The role of Schlafen 11 (SLFN11) as a predictive biomarker for targeting the DNA damage response. Br. J. Cancer.

[27] Stewart C.A., Tong P., Cardnell R.J., Sen T., Li L., Gay C.M., Masrorpour F., Fan Y., Bara R.O., Feng Y. (2017). Dynamic variations in epithelial-to-mesenchymal transition (EMT), ATM, and SLFN11 govern response to PARP inhibitors and cisplatin in small cell lung cancer. Oncotarget.

[28] Wang R., Cong Y., Li M., Bao J., Qi Y., Zhang J.Z.H. (2020). Molecular Mechanism of Selective Binding of NMS-P118 to PARP-1 and PARP-2: A Computational Perspective. Front. Mol. Biosci..

[29] Heym P.-P., Brandt W., Wessjohann L.A., Niclas H.-J. (2012). Virtual screening for plant PARP inhibitors—What can be learned from human PARP inhibitors?. J. Cheminformatics.

[30] Murai J., Huang S.N., Das B.B., Renaud A., Zhang Y., Doroshow J.H., Ji J., Takeda S., Pommier Y. (2012). Differential trapping of PARP1 and PARP2 by clinical PARP inhibitors. Cancer Res..

[31] Slade D. (2020). PARP and PARG inhibitors in cancer treatment. Genes Dev..

[32] Eikesdal H.P., Yndestad S., Elzawahry A., Llop-Guevara A., Gilje B., Blix E.S., Espelid H., Lundgren S., Geisler J., Vagstad G. (2021). Olaparib monotherapy as primary treatment in unselected triple negative breast cancer. Ann. Oncol. Off. J. Eur. Soc. Med. Oncol..

[33] Ryu H., Ahn J., Choi H.K. (2017). Novel Benzamide Derivatives: Synthesis and Bioactivity as Potent PARP-1 Inhibitors. Bull. Korean Chem. Soc..

[34] Berger R., Wessjohann L., Gonzalez Ceballos L., Heym P.P. (2024). Quinazolinones as Phytoeffectors.

[35] Berger R. Design, Synthesis and Evaluation of Drought Stress Tolerance-Inducing Compounds. Ph.D. Thesis.

[36] Menear K.A., Adcock C., Boulter R., Cockcroft X., Copsey L., Cranston A., Dillon K.J., Drzewiecki J., Garman S., Gomez S. (2008). 4-[3-(4-Cyclopropanecarbonylpiperazine-1-carbonyl)-4-fluorobenzyl]-2H-phthalazin-1-one: A Novel Bioavailable Inhibitor of Poly(ADP-ribose) Polymerase-1. J. Med. Chem..

[37] Maksimainen M.M., Murthy S., Sowa S.T., Galera-Prat A., Rolina E., Heiskanen J.P., Lehtiö L. (2021). Analogs of TIQ-A as inhibitors of human mono-ADP-ribosylating PARPs. Bioorganic Med. Chem..

[38] Griffin R.J., Srinivasan S., Bowman K., Calvert A.H., Curtin N.J., Newell D.R., Pemperton L.C., Golding B.T. (1998). Resistance-modifying agents. 5. Synthesis and biological properties of quinazolinone inhibitors of the DNA repair enzyme poly(ADP-ribose) polymerase (PARP). J. Med. Chem..

[39] Kulkarni S.S., Singh S., Shah J.R., Low W.-K., Talele T.T. (2012). Synthesis and SAR optimization of quinazolin-4(3H)-ones as poly(ADP-ribose)polymerase-1 inhibitors. Eur. J. Med. Chem..

[40] Rudolph J., Jung K., Luger K. (2022). Inhibitors of PARP: Number crunching and structure gazing. Proc. Natl. Acad. Sci. USA.

[41] Tutt A.N.J., Lord C.J., McCabe N., Farmer H., Turner N., Martin N.M., Jackson S.P., Smith G.C.M., Ashworth A. (2005). Exploiting the DNA repair defect in BRCA mutant cells in the design of new therapeutic strategies for cancer. Cold Spring Harb. Symp. Quant. Biol..

[42] Murai J., Feng Y., Yu G.K., Ru Y., Tang S.-W., Shen Y., Pommier Y. (2016). Resistance to PARP inhibitors by SLFN11 inactivation can be overcome by ATR inhibition. Oncotarget.

[43] Lok B.H., Gardner E.E., Schneeberger V.E., Ni A., Desmeules P., Rekhtman N., de Stanchina E., Teicher B.A., Riaz N., Powell S.N. (2017). PARP Inhibitor Activity Correlates with SLFN11 Expression and Demonstrates Synergy with Temozolomide in Small Cell Lung Cancer. Clin. Cancer Res. Off. J. Am. Assoc. Cancer Res..

[44] Krishnakumar R., Kraus W.L. (2010). The PARP Side of the Nucleus: Molecular Actions, Physiological Outcomes, and Clinical Targets. Mol. Cell.

[45] Banasik M., Komura H., Shimoyama M., Ueda K. (1992). Specific inhibitors of poly(ADP-ribose) synthetase and mono(ADP-ribosyl)transferase. J. Biol. Chem..

[46] Cockcroft X., Dillon K.J., Dixon L., Drzewiecki J., Kerrigan F., Loh V.M., Martin N.M.B., Menear K.A., Smith G.C.M. (2006). Phthalazinones 2: Optimisation and synthesis of novel potent inhibitors of poly(ADP-ribose)polymerase. Bioorganic Med. Chem. Lett..

[47] Johannes J.W., Almeida L., Daly K., Ferguson A.D., Grosskurth S.E., Guan H., Howard T., Ioannidis S., Kazmirski S., Lamb M.L. (2015). Discovery of AZ0108, an orally bioavailable phthalazinone PARP inhibitor that blocks centrosome clustering. Bioorganic Med. Chem. Lett..

[48] Coussens N.P., Braisted J.C., Peryea T., Sittampalam G.S., Simeonov A., Hall M.D. (2017). Small-Molecule Screens: A Gateway to Cancer Therapeutic Agents with Case Studies of Food and Drug Administration-Approved Drugs. Pharmacol. Rev..

[49] Dai X., Cheng H., Bai Z., Li J. (2017). Breast Cancer Cell Line Classification and Its Relevance with Breast Tumor Subtyping. J. Cancer.

[50] Ruberte A.C., Plano D., Encío I., Aydillo C., Sharma A.K., Sanmartín C. (2018). Novel selenadiazole derivatives as selective antitumor and radical scavenging agents. Eur. J. Med. Chem..

[51] Berman H.M., Westbrook J., Feng Z., Gilliland G., Bhat T.N., Weissig H., Shindyalov I.N., Bourne P.E. (2000). The Protein Data Bank. Nucleic Acids Res..

[52] Nottbohm A.C., Dothager R.S., Putt K.S., Hoyt M.T., Hergenrother P.J. (2007). A Colorimetric Substrate for Poly(ADP-Ribose) Polymerase-1, VPARP, and Tankyrase-1. Angew. Chem..

[53] Kanamori K.S., de Oliveira G.C., Auxiliadora-Martins M., Schoon R.A., Reid J.M., Chini E.N. (2018). Two Different Methods of Quantification of Oxidized Nicotinamide Adenine Dinucleotide (NAD+) and Reduced Nicotinamide Adenine Dinucleotide (NADH) Intracellular Levels: Enzymatic Coupled Cycling Assay and Ultra-performance LiquidChromatography (UPLC)-Mass Spectrometry. Bio-Protoc..

[54] Rao X., Huang X., Zhou Z., Lin X. (2013). An improvement of the 2ˆ(–delta delta CT) method for quantitative real-time polymerase chain reaction data analysis. Biostat. Bioinform. Biomath.

[55] Rahn J., Lennicke C., Kipp A.P., Müller A.S., Wessjohann L.A., Lichtenfels R., Seliger B. (2017). Altered protein expression pattern in colon tissue of mice upon supplementation with distinct selenium compounds. Proteomics.

[56] Feoktistova M., Geserick P., Leverkus M. (2016). Crystal Violet Assay for Determining Viability of Cultured Cells. Cold Spring Harb. Protoc..

[57] Seixas N., Ravanello B.B., Morgan I., Kaluđerović G.N., Wessjohann L.A. (2019). Chlorambucil Conjugated Ugi Dendrimers with PAMAM-NH_2_ Core and Evaluation of Their Anticancer Activity. Pharmaceutics.

[58] Sultani H.N., Morgan I., Hussain H., Roos A.H., Haeri H.H., Kaluđerović G.N., Hinderberger D., Westermann B. (2021). Access to New Cytotoxic Triterpene and Steroidal Acid-TEMPO Conjugates by Ugi Multicomponent-Reactions. Int. J. Mol. Sci..

[59] Morgan I., Wessjohann L.A., Kaluđerović G.N. (2022). In Vitro Anticancer Screening and Preliminary Mechanistic Study of A-Ring Substituted Anthraquinone Derivatives. Cells.

[60] Hwang K., Lee J.-H., Kim S.H., Go K.-O., Ji S.Y., Han J.H., Kim C.-Y. (2021). The Combination PARP Inhibitor Olaparib With Temozolomide in an Experimental Glioblastoma Model. In Vivo.

[61] Irwin C.P., Portorreal Y., Brand C., Zhang Y., Desai P., Salinas B., Weber W.A., Reiner T. (2014). PARPi-FL—A Fluorescent PARP1 Inhibitor for Glioblastoma Imaging. Neoplasia.

[62] Salinas B., Irwin C.P., Kossatz S., Bolaender A., Chiosis G., Pillarsetty N., Weber W.A., Reiner T. (2015). Radioiodinated PARP1 tracers for glioblastoma imaging. EJNMMI Res..

