# Supplementary material for: Identification and Characterization of Novel Inhibitors of Human Poly(ADP-Ribose) Polymerase-1
Source: Molecules. 2025 Jun 25;30(13):2728. doi: 10.3390/molecules30132728 (PMC12250713; doi:10.3390/molecules30132728)
Supplement: Supplementary file 1 [file molecules-30-02728-s001.zip › molecules-3580049-supplementary.pdf]

molecules

Supplementary information

Identification and characterization of novel inhibitors of human poly(ADP-ribose) polymerase-1

Ibrahim Morgan, Robert Rennert *, Robert Berger, Ahmed Hassanin, Mehdi D. Davari, Daniela Eisenschmidt-Bönn † and Ludger A. Wessjohann *

Department of Bioorganic Chemistry, Leibniz Institute of Plant Biochemistry, Weinberg 3, 06120 Halle (Saale), Germany; ibrahim.morgan@ipb-halle.de (I.M.); r_berger@outlook.de (R.B.); ahassanin@ipb-halle.de (A.H.); mehdi.davari@ipb-halle.de (M.D.D.); daniela.eisenschmidt-boenn@uk-halle.de (D.E.-B.)

* Correspondence: robert.rennert@ipb-halle.de (R.R.); wessjohann@ipb-halle.de (L.A.W.); Tel.: +49-(0)3-4555-821301 (L.A.W.)

† Current address: Section Information and Communication Technology, University Medicine Halle, Ernst-Grube-Straße 40, 06120 Halle (Saale), Germany.

Table S1: List of the compounds identified as potential hPARP-1 inhibitors by the *in silico* hPARP-1 docking and screening study.

Compound	Structure	Compound	Structure
1		2	
3		4	
5		6	
7		8	

9	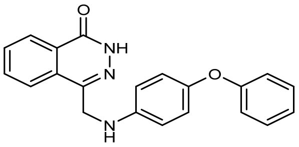	10	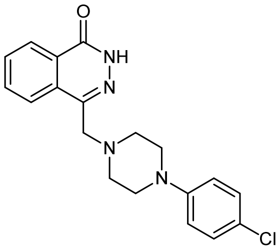
11	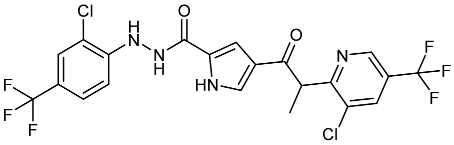	12	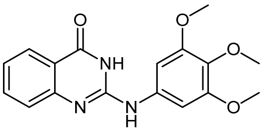
13	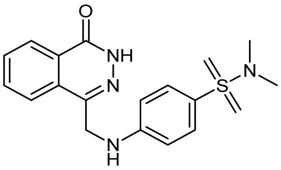	14	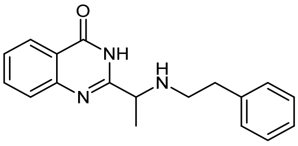
15	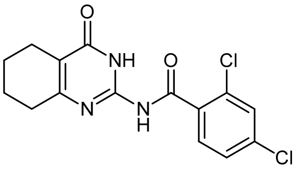	16	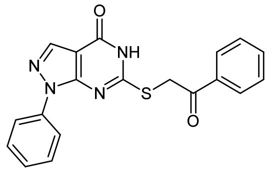
17	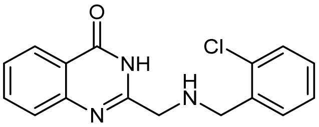	18	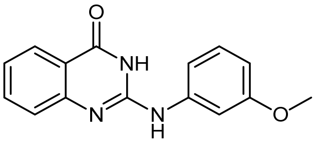
19	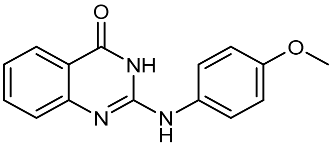	20	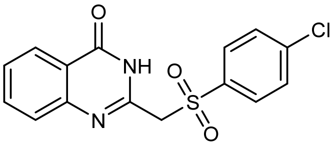
21	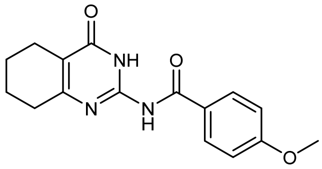	22	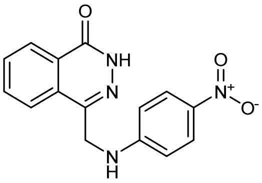
23	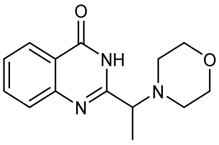	24	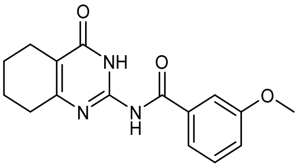
25	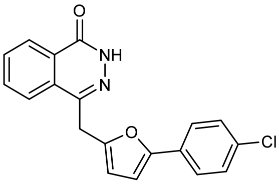	26	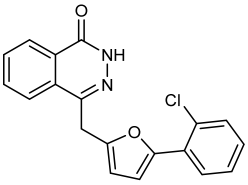
27	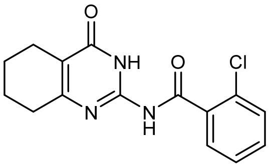	28	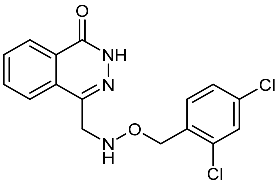

29	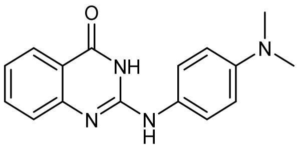	30	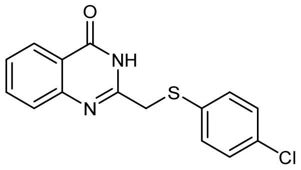
31	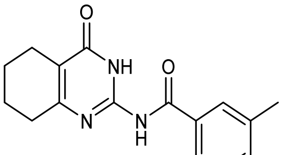	32	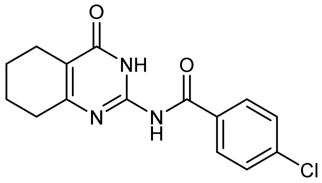
33	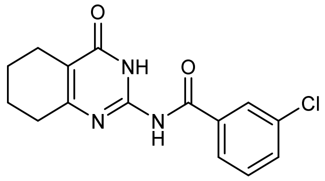	34	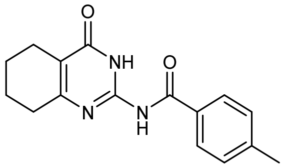
35	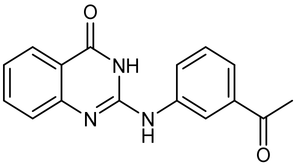	36	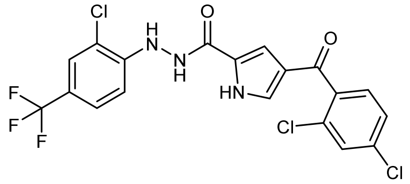
37	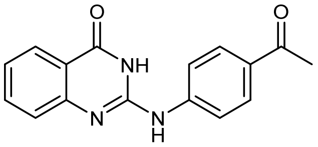	38	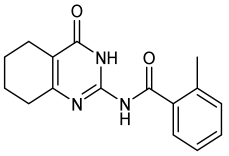
39	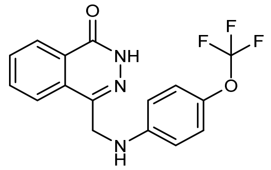	40	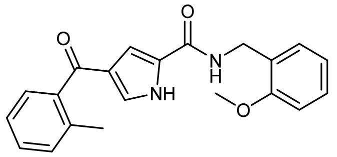
41	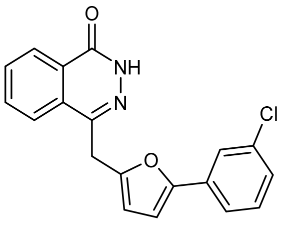	42	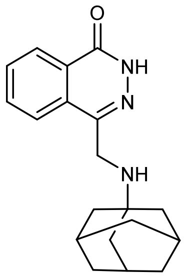
43	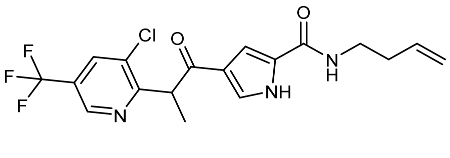	44	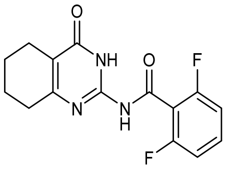
45	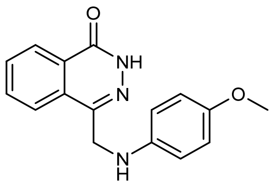	46	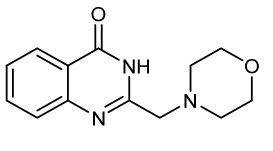

47	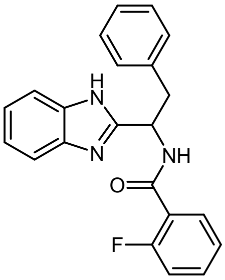	48	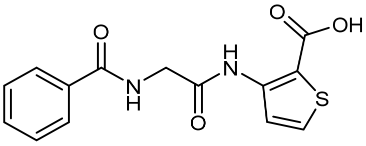
49	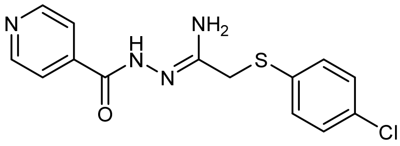	50	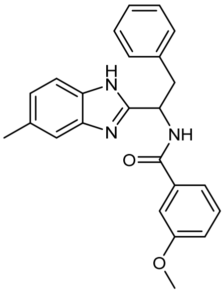
51	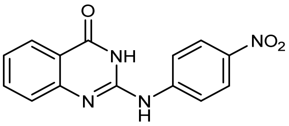	52	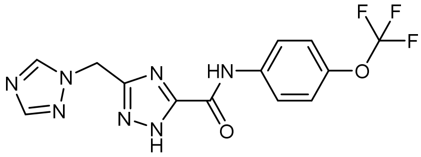
53	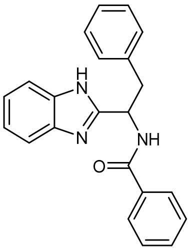	54	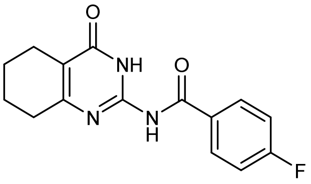
55	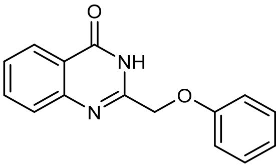	56	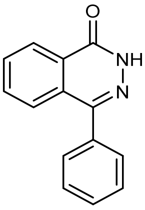
57	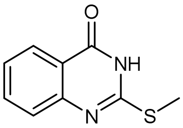	58	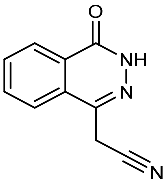
59	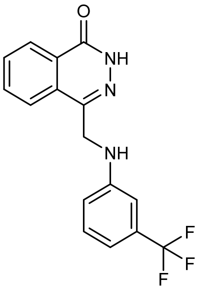	60	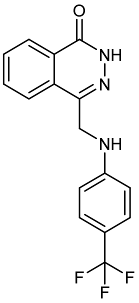

61	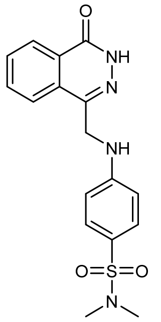	62	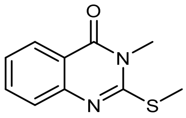
63	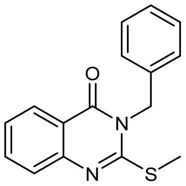	64	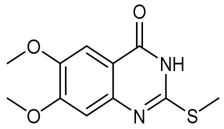
65	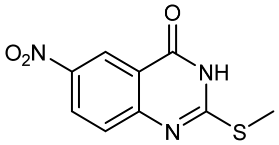	66	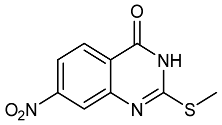
67	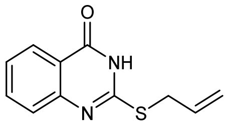	68	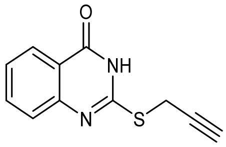
69	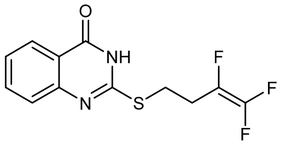		

Scheme S1: Synthesis and analytical data of compound **57**

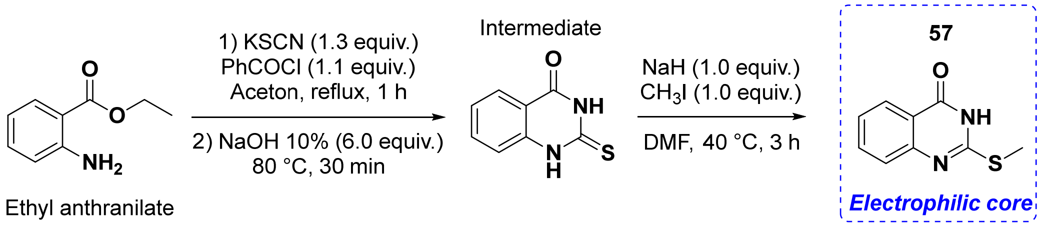

Compound 57 was synthesized in a two-stepped reaction. To a well stirred solution of potassium thiocyanate (6.5 g, 65 mmol) in 75 ml of dry acetone was added dropwise benzoyl chloride (6.41 ml, 55.0 mmol) and the mixture was heated to reflux (oil bath). After 15 min, ethyl anthranilate (7.47 ml, 50.0 mmol) was added dropwise, and the mixture was refluxed for further 60 min. The mixture was cooled down, poured on ice water (350 ml), and stirred for 30 min. The precipitate was filtered off, washed with water, and added portion wise to preheated (80 °C) sodium hydroxide solution (125 ml, 10% w/v) with stirring. After complete dissolution, the mixture was stirred for further 30 min, cooled to room temperature, and then poured on ice. The pH was adjusted to 2-3 with HCl (conc.) and the mixture was stirred for further 30 min. Finally, the intermediate product was filtered off, washed with water, and recrystallized from DMF/water.

Subsequently, to a stirred suspension of sodium hydride (1.60 g, 40 mmol, 60% suspension in mineral oil) in dry DMF (60 ml) at room temperature (water bath), the intermediate product (7.13 g, 40 mmol) was slowly added portion wise. Upon complete dissolution, methyl iodide (2.49 ml, 40 mmol) was added dropwise, and the mixture was stirred for 3 h at 40 °C. After that, the mixture was cooled down to room temperature, poured on ice, and stirred for 30 min. The product was filtered off and washed with water and diethyl ether. Following recrystallization from DMF/water the pure compound 57 was obtained.

57

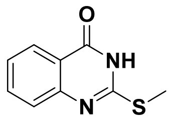

Chemical Formula: $C_9H_8N_2OS$
Exact Mass: 192,04
Molecular Weight: 192,24

Compound **57**, white needles, 6.3 g, 82% yield.

^{1}H NMR (400 MHz, DMSO-*d*$_6$) δ 12.50 (s, 1H), 7.96 (d, *J* = 7.9 Hz, 1H), 7.68 (t, *J* = 7.7 Hz, 1H), 7.46 (d, *J* = 8.2 Hz, 1H), 7.34 (t, *J* = 7.7 Hz, 1H), 2.43 (s, 3H).

^{13}C NMR (100 MHz, DMSO) δ 161.08, 156.27, 148.43, 134.55, 126.00, 125.96, 125.53, 119.91, 12.71.

ESI-HRMS, calculated for $C_9H_7N_2OS$: 191.0285 [M−H]$^-$, found: m/z 191.0283.

Compound **57** – NMR data:

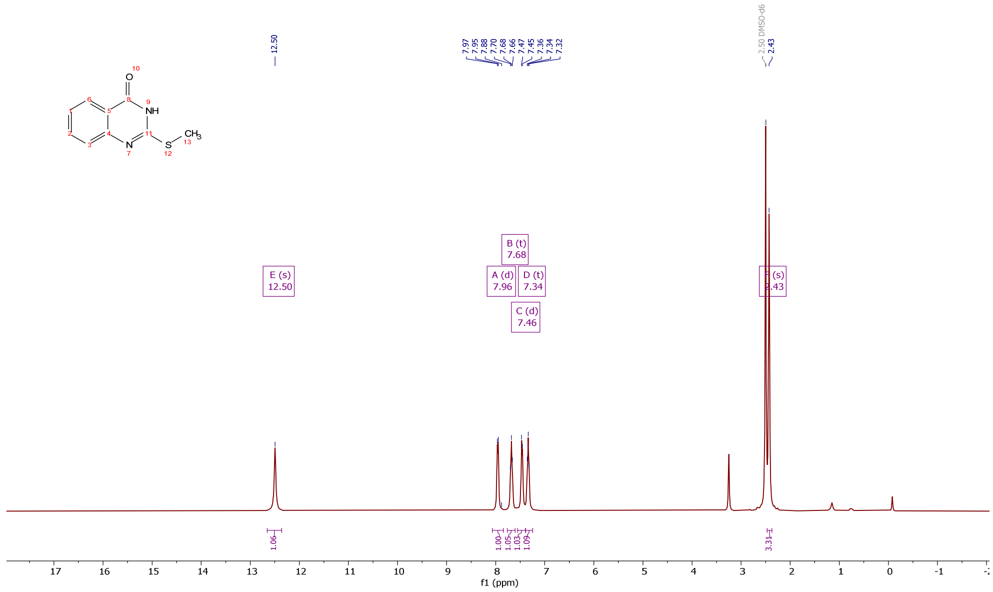

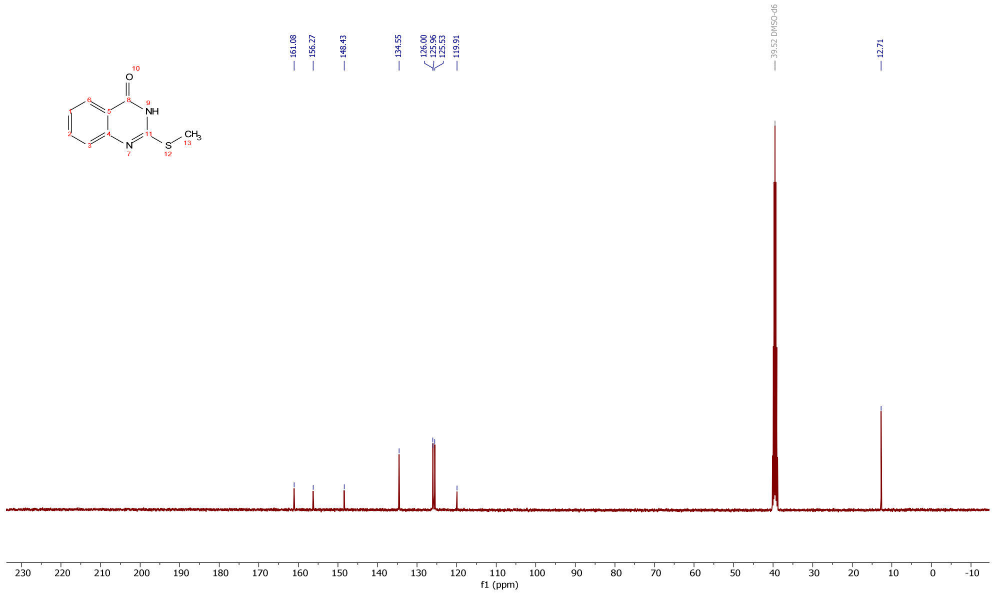

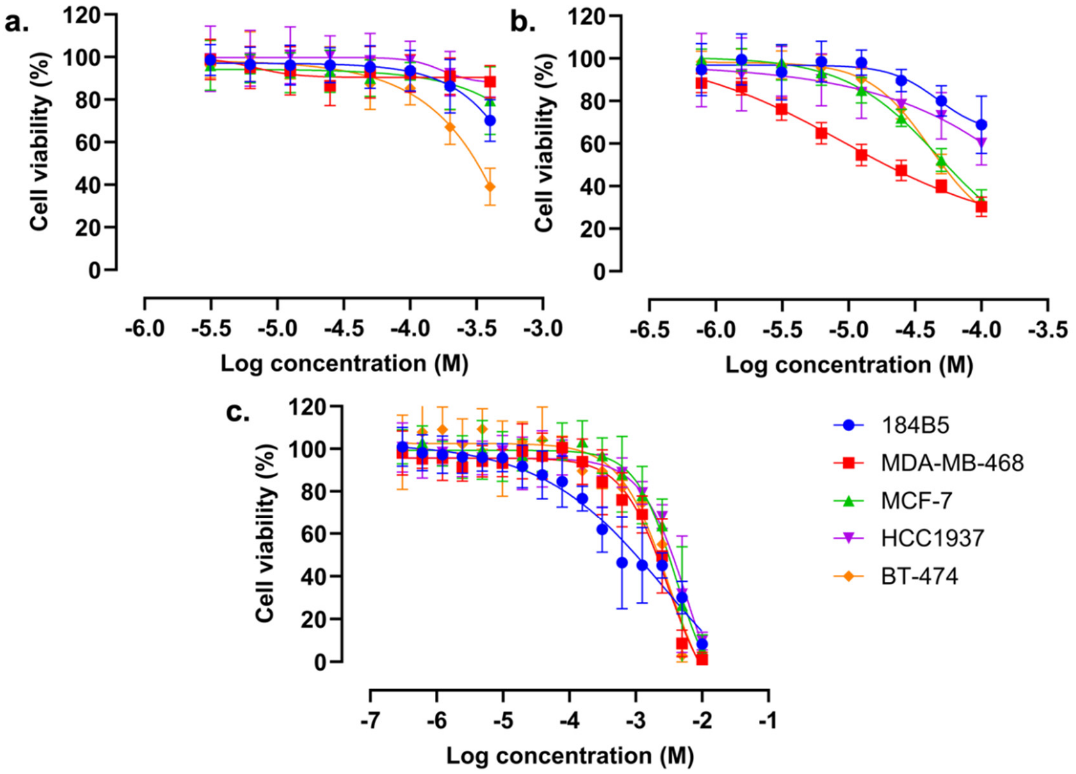

Figure S1: Cell viability dose-response curves of the breast cell lines under investigation upon 72 h treatment with **a,** compound **57**; **b,** OLP, and **c,** TMZ. Cell viability was determined by using crystal violet (CV) assay. Results are presented as the mean ± standard deviation. The mean is calculated from two biological replicates, each comprising three technical replicates.

Table S2: Summary of the calculated IC_{50} values (given in mM) of TMZ, TMZ combined with 100 µM of compound **57**, and TMZ combined with 1.56 µM of OLP as tested against the breast cell lines. Cells were treated for 72 h, and cell viability was determined by using the CV assay.

Treatment	184B5	MDA-MB-468	MCF-7	HCC1937	BT-474
TMZ	0.98 ± 0.82	2.06 ± 0.79	3.44 ± 1.72	3.74 ± 1.5	2.21 ± 0.01
TMZ + compound 57	0.32 ± 0.07	2.77 ± 0.25	2.23 ± 0.74	5.66 ± 0.32	4.87 ± 6.42
TMZ + OLP	4.95 ± 3.9	0.13 ± 0.04	2.85 ± 2.08	3.66 ± 1.04	5.05 ± 0.56

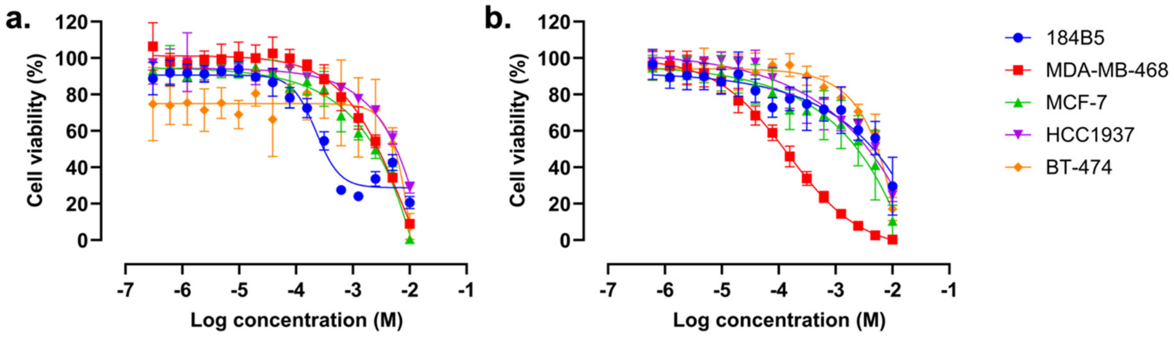

Figure S2: Dose-dependent curves of several breast cell lines. The cell lines were treated with several concentrations of TMZ combined with IC_{10} of **a,** compound 57 and **b,** OLP for 72 h, and the viability was determined using CV assay. Results are presented as the mean ± standard deviation. The mean is calculated from two biological replicates, each comprising three technical replicates.

Table S3: Sequences of the qPCR primers used in the gene expression study.

Gene name	Sense sequence (5′-3′)	Antisense sequence (5′-3′)
GAPDH	TTG CCA TCA ATG ACC CCT TCA	CGC CCC ACT TGA TTT TGG A
SLFN-11	CCCGATAACCTTCACACTCA	CTGATCATGCAAGCATAGCC
hPARP-1	ATT TCG CTG ACA TGG TCT CC	ACA TGT TTC CAA GGG CAA CT

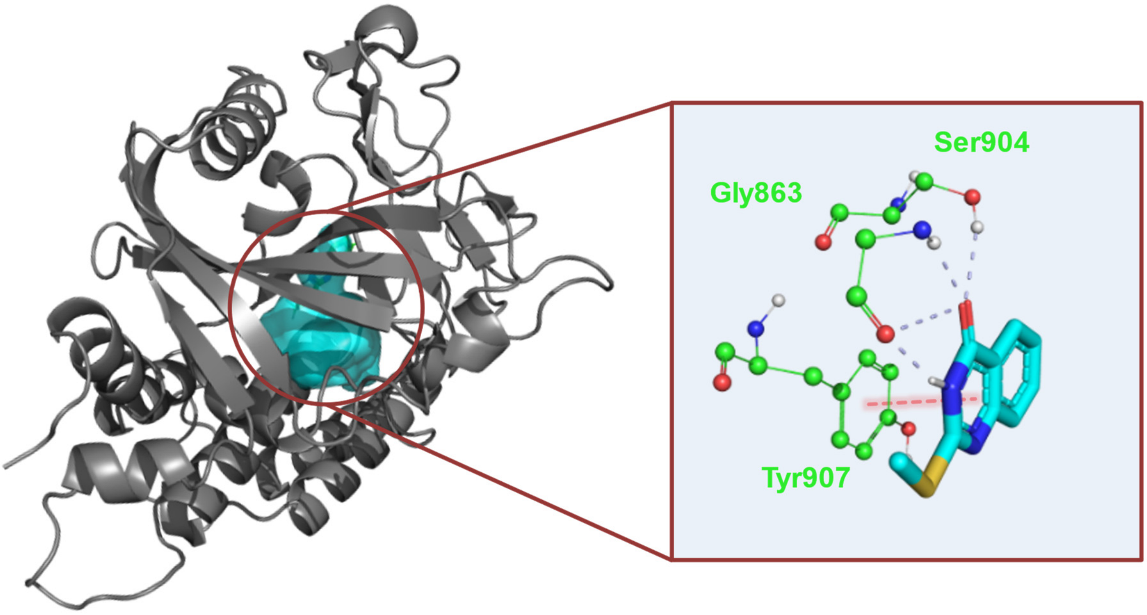

Figure S3: The structure of the ART subdomain of hPARP-1 (PDB code: 3GJW) interacting with the newly identified hPARP inhibitor compound 57 (cyan). Closeup view of the active site shows the hydrogen bond interactions with Ser904 and Gly863, and π-π stacking interactions with Try907.

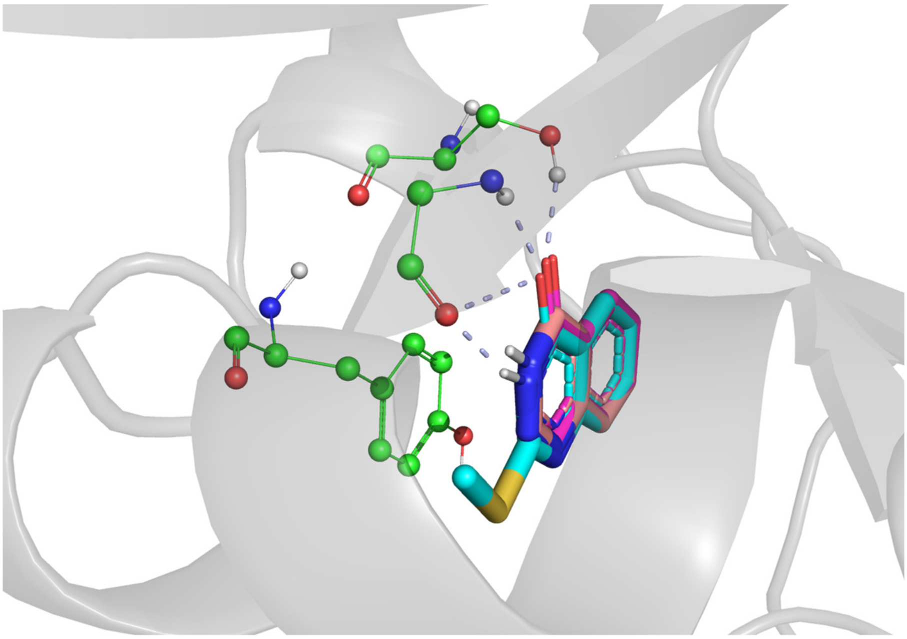

Figure S4: The structure of the ART subdomain of hPARP-1 active site with the overlayed PARP inhibitor compound **57 (**cyan), Quinazolin-4(*3H*)-one (orange) and Phthalazin-1(*2H*)-one (pink).

Table S4: Binding energies of compound **57**, Olaparib, and their underlying core structures interacting with the ART domain of hPARP-1 from molecular docking using MOE 2022.2 (Molecular Operating Environment (MOE), 2022.02 Chemical Computing Group ULC, Montreal, QC, Canada) software.

Compound name	Binding energy (kcal/mol)
Compound **57**	-5.90
Quinazolin-4(*3H*)-one	-5.31
Olaparib	-8.16
Phthalazin-1(*2H*)-one	-5.35
